# Guiding catalytic CO_2_ reduction to ethanol with copper grain boundaries†

**DOI:** 10.1039/d3sc02647g

**Published:** 2023-06-22

**Authors:** Dongfang Cheng, Gong Zhang, Lulu Li, Xiangcheng Shi, Shiyu Zhen, Zhi-Jian Zhao, Jinlong Gong

**Affiliations:** a Key Laboratory for Green Chemical Technology of Ministry of Education, School of Chemical Engineering and Technology, Tianjin University Tianjin 300072 China jlgong@tju.edu.cn; b Collaborative Innovation Center of Chemical Science and Engineering (Tianjin) Tianjin 300072 China; c Joint School of National University of Singapore and Tianjin University, International Campus of Tianjin University Binhai New City Fuzhou 350207 China; d Haihe Laboratory of Sustainable Chemical Transformation Tianjin 300192 China

## Abstract

The grain boundaries (GBs) in copper (Cu) electrocatalysts have been suggested as active sites for CO_2_ electroreduction to ethanol. Nevertheless, the mechanisms are still elusive. Herein, we describe how GBs tune the activity and selectivity for ethanol on two representative Cu-GB models, namely Cu∑3/(111) GB and Cu∑5/(100) GB, using joint first-principles calculations and experiments. The unique geometric structures on the GBs facilitate the adsorption of bidentate intermediates, *COOH and *CHO, which are crucial for CO_2_ activation and CO protonation. The decreased CO–CHO coupling barriers on the GBs can be rationalized *via* kinetics analysis. Furthermore, when introducing GBs into Cu (100), the product is selectively switched from ethylene to ethanol, due to the stabilization effect for *CH_3_CHO and inapposite geometric structure for *O adsorption, which are validated by experimental trends. An overall 12.5 A current and a single-pass conversion of 5.18% for ethanol can be achieved over the synthesized Cu-GB catalyst by scaling up the electrode into a 25 cm^2^ membrane electrode assembly system.

## Abstract 2

This paper describes how GBs facilitate the generation of C_2+_ alcohols. An overall 12.5 A current and a single-pass conversion of 5.18% for ethanol can be achieved on the Cu-GB catalyst by scaling up the electrode into a 25 cm^2^ MEA system.

## Graphical abstract

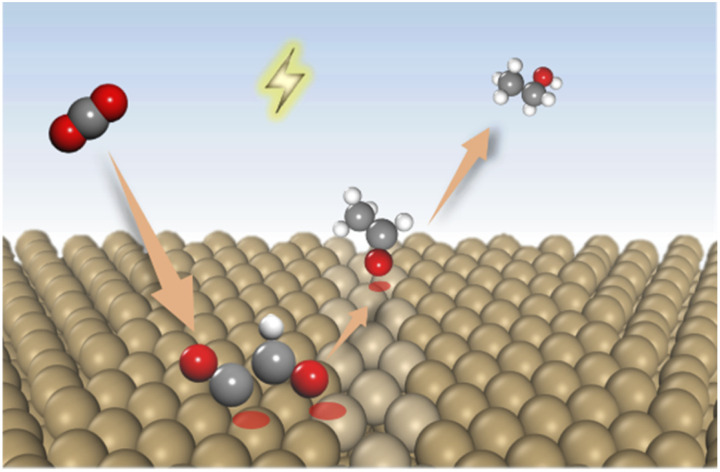

## Introduction

Electrochemical CO_2_ reduction (CO_2_R) to valuable multi-carbon (C_2+_) products offers a promising solution for alleviating the climate change crisis and storing renewable energy.^1–8^ Copper (Cu) is by far the only metal that can reduce CO_2_ to C_2+_ products.^9–14^ To date, many strategies, like oxide-derived processes,^15–17^ surface morphology regulation,^18,19^ nonmetal doping,^20,21^ and alloying,^22,23^ have been proposed to modify Cu-based catalysts to further improve the activity and selectivity of CO_2_R to C_2+_ products, especially ethanol. From the point of view of the chemical industry, ethanol is one kind of liquid fuel with high energy density, which has a relatively high market price and is considered an important intermediate in chemical synthesis.^24^ However, most Cu-based catalysts generate more ethylene than ethanol. Although many efforts have been made to increase the faradaic efficiency of ethanol production and several great achievements have been made,^20,24–29^ there is still a lack of sufficient current density of ethanol or the catalysts suffer from low conversion efficiency in ethanol production.

Recently, grain boundary (GB) engineering has been considered a viable method to control the activity and selectivity for the CO_2_R reaction.^15,16^ Kanan *et al.* proposed that the grain boundaries (GBs) serve as strengthened CO binding sites on Cu catalysts, which are crucial for CO_2_R to C_2+_ products.^30^ Later, a linear correlation between the density of GBs and CO reduction activity was found over Cu nanoparticles on carbon nanotubes.^31^ In our recent work we synthesized grain-boundary-rich metallic copper and obtained 70% selectivity for ethylene and ethanol. Specifically, the ratio of ethanol to ethylene was enhanced from 0.49 to 0.85.^32^ These experimental results strongly suggest that Cu GBs have a positive effect on the production of C_2+_ oxygenates. However, to date, the mechanism of Cu GBs regulation on C_2+_ selectivity remains elusive. To illustrate this relationship, two fundamental problems should be addressed: (1) can the Cu GBs promote the rate-determining step, C–C coupling, for C_2+_ products formation? (2) How will the Cu GBs regulate the bifurcation pathways towards ethanol?

In this work, we employed first principles calculations to explore how Cu GBs tune the activity and selectivity of CO_2_R to ethanol on representative Cu∑3/(111) GB and Cu∑5/(100) GB models. We found that the undercoordinated sites with relatively longer Cu–Cu bond length on GBs together have strong impacts on the binding strength of bidentate adsorbates, *COOH and *CHO, which are conducive to the initial activation of CO_2_ and further CO reduction, respectively. Kinetics analysis found that CO–CHO coupling is the preferred reaction type at the applied potential; meanwhile, the barriers can be reduced on (111)-GBs compared to those on (111) terrace sites. Interestingly, when carrying out GB engineering on Cu (100), it is intriguing to observe a distinct modulation in the reaction pathway, leading to a notable transition from ethylene synthesis to ethanol production. Based on the collected information, we proposed our strategy, introducing low coordinated sites into the Cu (100) surface, to improve the selectivity for ethanol over Cu-based catalysts. Furthermore, considering Cu GBs have great potential to produce ethanol in theory, we then scaled up the electrode to a 25 cm^2^ membrane electrode assembly system and obtained a 12.5 A total current and single-pass conversion of 5.18% for ethanol over the synthesized Cu-GB catalyst.

## Results and discussion

### Mechanism exploration on CuGB

**Fig. 1:**
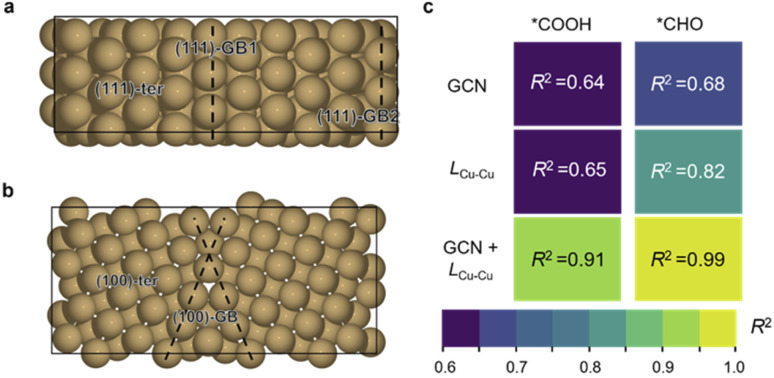
Top view of (a) the Cu ∑3/(111) grain boundary and (b) Cu ∑5/(100) grain boundary. The GB areas are labeled by black dashed lines. (c) Linear regression analysis of adsorption energies of *COOH and *CHO with GCN and Cu–Cu bond length (*L*_Cu–Cu_). The figure shows the *R*^2^ coefficient obtained by linearly correlating the adsorption energies of *COOH and *CHO with the GCN, Cu–Cu bond length (*L*_Cu–Cu_) and both by multi-linear regression.

Herein, Cu∑3/(111) GB and Cu∑5/(100) GB models were built according to coincidence site lattice theory^33^ (Fig. 1a and b, details are shown in the ESI†). Five active sites were chosen from these two models to compare the catalytic activity: GB1 and GB2 on ∑3/(111) GB (referred to as 111-GB1 and 111-GB2, respectively), GB on ∑5/(100) (referred to as 100-GB), and terrace sites (referred to as 111-ter and 100-ter, respectively) (Fig. 1a and b). It is impractical to model all kinds of GBs, and we expect that these five sites can in general illuminate how GB structures tune the catalytic performance compared to that of terrace sites.

Two appropriate scaling relationships between the adsorption energy of *COOH and *CHO with *CO are observed (ESI Fig. S1a†), which is consistent with previous research.^34,35^ We found that on GBs, *COOH and *CHO adsorption are apparently enhanced compared to the terrace sites, especially in (111)-GB2 and (100)-GB sites, which fall below their scaling relationships. The stronger *COOH adsorption indicates that CO_2_ is more likely to be activated, which means GBs show positive impacts on the initial activation of CO_2_ to form a stable *COOH intermediate. We notice that on the (111)-ter site, *CO tends to desorb to form gaseous CO, while on the other sites, *CO is favorable to be hydrogenated to *CHO. *CHO, therefore, is the major species formed *via* protonation of *CO on these sites, given that the reaction free energy (Δ*G*) of further protonation of *CO to *CHO is much lower on GBs compared to *COH (ESI Fig. S1b†). Further, we explored the reaction barriers of this proton–electron transfer process on GBs. The kinetic barriers of *CO to *CHO are 0.55 eV, 0.52 eV and 0.35 eV on (111)-GB1, (111)-GB2 and (100)-GB (ESI Fig. S2†), respectively. All these three sites show facile kinetic barriers, lower than 0.75 eV, corresponding to a turnover frequency of 1 per s per site at room temperature.^36^

**Fig. 2:**
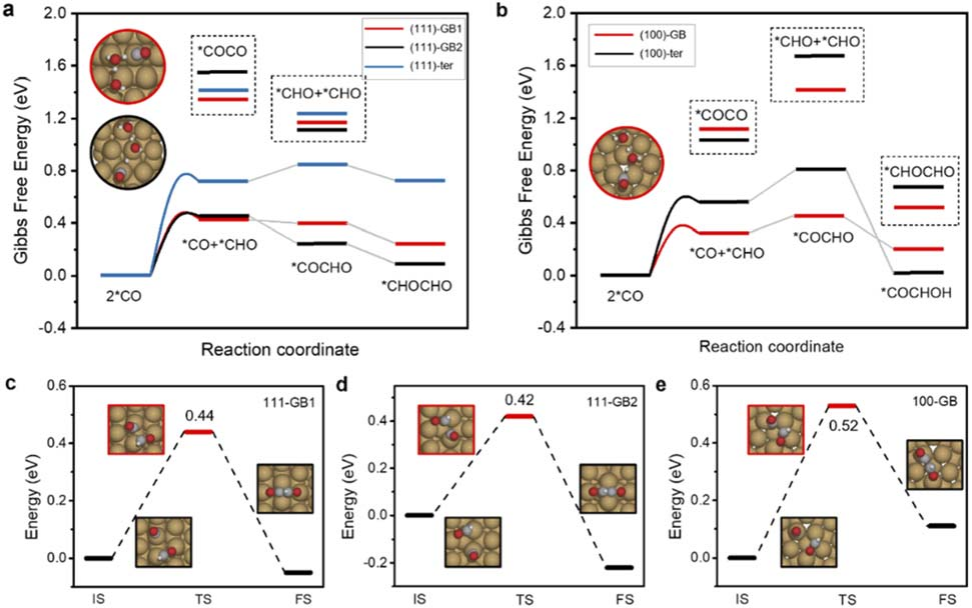
Free energy diagrams of C–C coupling for (a) ∑3/(111) GBs and (b) ∑5/(100) GBs. Reaction barriers of CHO–CO coupling for (c) 111-GB1, (d) 111-GB2 and (e) 100-GB.

We then investigated the origin of the enhanced adsorption of these two intermediates. General coordination number (GCN) is a universal description which has been widely used to explain the structure–adsorption behavior.^37,38^ We thus tried to scale the adsorption energy of *COOH and *CHO to GCN. However, the results of the direct fitting are not satisfactory (*R*^2^ = 0.64 and 0.68) (Fig. 2c and ESI Fig. S3†), which implies the coordination environment alone cannot fully explain these behaviors. Huang *et al.*^39^ and Xin *et al.*^40^ proposed that the bond length of the surface atom can change the lattice strains and further influence the adsorption of intermediates. In addition, the adsorption modes of *COOH and *CHO are both bidentate, where C and O are each bonded to adjunct Cu atoms. Based on the above analysis, we believe that the Cu–Cu bond length (*L*_Cu–Cu_) on GBs plays an important role in the adsorption of *COOH and *CHO (Fig. 2c and ESI Fig. S4†). We thus combine the GCN and *L*_Cu–Cu_ to perform the regression analysis with the adsorption energy. Surprisingly, the correlation coefficients (*R*^2^) significantly increase if these two factors are both taken into consideration (*R*^2^ = 0.91 and 0.99) for *COOH and *CHO, respectively (Fig. 2c and ESI Fig. S5†). This indicates that GBs offer the undercoordinated surface sites and relatively longer Cu–Cu bond length, together effectively stabilizing two important bidentate adsorbates, *COOH and *CHO, which are beneficial for CO_2_ activation and further protonation of *CO. We further performed the crystal orbital Hamilton population (COHP) analysis and found that on GB sites, the interactions between the adsorbates and the surface are much stronger (ESI Fig. S6†). The adsorption energies of *COOH and *CHO exhibit positive correlations with the values of ICOHP (ESI Fig. S7†). Kim *et al.* proposed that the broken local spatial symmetry near the GBs tunes the metal-to-adsorbate π-backbonding ability, thereby stabilizing the COOH intermediate.^41^ Projected density of states (PDOS) shows that *CHO intermediates showed a higher degree of overlap with Cu d-orbitals than that on terrace sites between −5.5 and −7.5 eV under the Fermi level (ESI Fig. S8†). The higher overlap suggests that GBs can significantly stabilize the key *CHO intermediate.

It can be found that *CO adsorption is strengthened on GBs compared to that on their terrace sites. This phenomenon is consistent with our previous *in situ* attenuated total reflection surface-enhanced infrared absorption spectroscopy (ATR-SEIRAS) results^32^ and former observation over OD-Cu catalysts of stronger adsorption of CO on the GB-Cu surface.^30^ The stronger CO binding strength indicates that on GBs, CO coverage is much higher. Liu *et al.* found that C_2_ activity is more affected than C_1_ since it has a second-order dependence on *CO coverage.^42^ Therefore, GBs may have the ability to accelerate the C–C coupling to C_2+_ products.

**Fig. 3:**
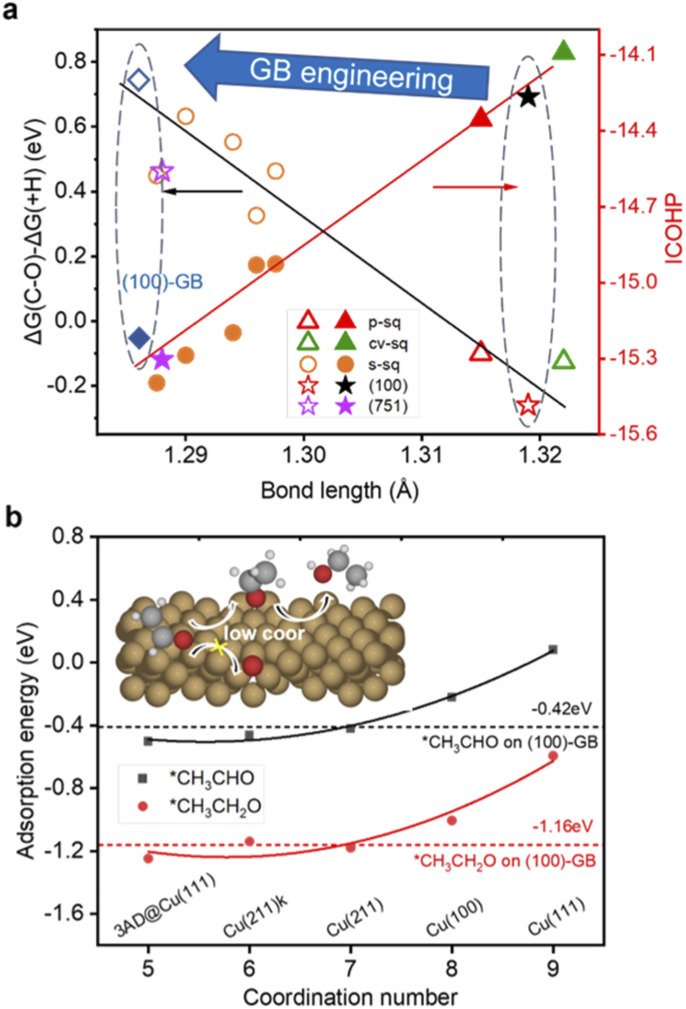
(a) Black: The relationship between the bond length of C–O in adsorbed CH_2_CHO and the difference of Gibbs free reaction energy of C–O scission (Δ*G*(C–O)) and hydrogenation (Δ*G*(+H)). Red: The relationship between the bond length of C–O in adsorbed CH_2_CHO and the ICOHP of the C–O bond. See ref. 24. (b) The relationships between the coordination number and the adsorption energy of *CH_3_CHO and *CH_3_CH_2_O. The red and black dashed horizontal lines represent the adsorption energy of *CH_3_CHO and *CH_3_CH_2_O on the (100)-GB site (−0.42 eV and −1.16 eV). The inset shows the general mechanism of ethanol production over the (100)-GB site.

The current consensus regarding C–C coupling over Cu surfaces is that two *CO undergo dimerization, followed by protonation to a *COCOH or *COCHO intermediate.^9^ Our calculations show that CO protonation to *COH is difficult neither on terrace sites nor on GB sites, which show at least 0.2 eV higher reaction energy than *CHO formation. Thus, we exclude the coupling form of *CO–COH or *COH–*COH (ESI Fig. S1†). We found that *CO protonated to *CHO, and the *CO and *CHO coupling is thermodynamically most favorable on GBs. Meanwhile, the coupling of two adsorbed *CO species requires quite high uphill reaction energies either on the GB sites or on the terrace sites (Fig. 3a and b). Thus, *CO couples with *CHO are much more facile than 2*CO dimerization. In addition, the *COCHO formation energy by coupling of *CO and *CHO is lower than hydrogenation of the other co-adsorbed *CO, suggesting that the coupling of 2*CHO is thermodynamically unfavorable.

From the kinetic point of view, the reaction barriers for *CO and *CHO coupling on (111)-GB1 (0.44 eV) and (111)-GB2 (0.42 eV) are much lower than that on (111)-ter (1.10 eV), whereas a slight decrease can be seen on (100)-GB (0.52 eV) with respect to (100)-ter (0.62 eV). The reaction barriers for dimerization of two *CO species are extremely high (>1 eV) on these sites in the vacuum. Considering that the *COCO intermediate with large dipole moment is highly sensitive to the interface electric field and solvent environment,^9^ we established one layer of charged water to take the solvent and electric field effect into consideration. Table 1 clearly shows that two *CO coupling barriers can be much more reduced under the charged water; however, they are still larger than *CO–*CHO coupling and CO protonation to *CHO (0.53 eV, 0.52 eV, 0.35 eV on (111)-GB1, 111-GB2, and 100-GB, respectively). Alexis T. Bell *et al.* proposed that with the increase of overpotential, the reaction barrier of *CO protonation to *CHO continuously decreases, while the tendency of *CO–*CO coupling is the opposite.^43^ Our previous experiment showed that the overpotential needed to be as high as −1.0 V *vs.* RHE to achieve the high C_2+_ activity on GB-rich copper catalysts.^32^ Considering the surmountable barriers of *CO protonation calculated above, we can conclude that at relatively high overpotential, *CO protonation to *CHO is preferred compared to *CO–*CO coupling on GBs. This indicates that the (111) surface, which is considered as one of the least active facets for C_2+_ products generation, can promote C–C coupling and improve the catalytic activity for C_2+_ products when introducing GBs. Though the coupling barrier is not obviously decreased when introducing GBs on the (100) surface, the enhanced *CO and *CHO adsorption will increase the coverages of coupling species and further improve the reaction rate for C_2+_ products.

**Table 1:** Reaction barriers of 2*CO coupling and *CO–*CHO coupling on different sites. All values are in eV

Site	*CO–CO (in a vacuum)	*CO–CO (with charged water)	*CO–CHO
(111)-GB1	1.35	0.53	0.44
(111)-GB2	1.55	1.13	0.42
(111)-ter	1.42	0.85	1.10
(100)-GB	1.34	0.52	0.49
(100)-ter	1.34	0.49	0.62

The bifurcation pathway for ethylene and ethanol on different sites is another important issue we are concerned about, which determines the selectivity of the catalysts. *CH_2_CHO is considered as the selectivity-determining intermediate.^44^ Ethylene is produced *via* breaking the C–O bond while ethanol is generated from further protonation.^45,46^ The free energy diagrams (ESI Fig. S9†) reveal that (111)-GB1 and (111)-GB2 have preference for ethanol production. Further protonation of *CH_2_CHO to *CH_3_CHO intermediates (Δ*G*(+H)) is exothermic on both sites. Reaction free energies of scission of C–O (Δ*G*(C–O)) are 0.15 eV and 0.05 eV on (111)-GB1 and (111)-GB2, respectively. Although (111)-ter shows preference to the ethanol pathway as well (ESI Fig. S10†), unfavorable C–C dimerization prevents this site from generating abundant C_2+_ products. On the (100)-ter site, the ethylene pathway is more advantageous than the ethanol pathway, which is consistent with previous experimental and theoretical works which suggest that Cu (100) is an active site for ethylene formation. When GB engineering is performed on the (100) surface, Δ*G*(C–O) and Δ*G*(+H) are 0.49 eV and −0.25 eV. At this point, the balance is tilted towards producing ethanol, rather than ethylene.

**Fig. 4:**
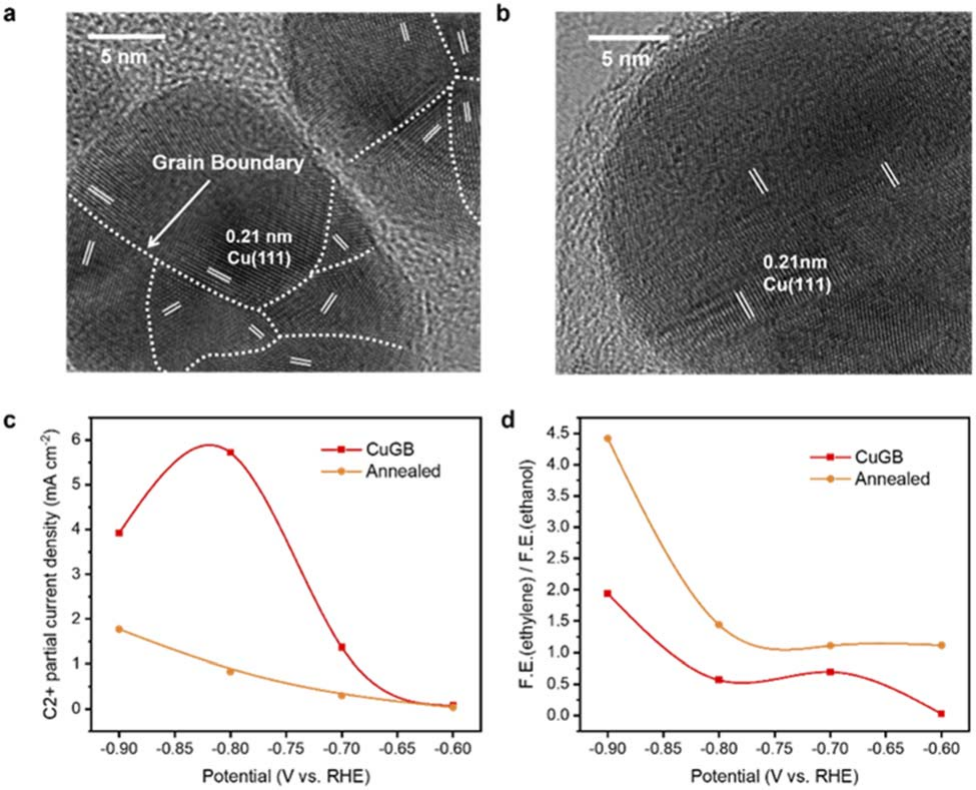
HRTEM images of (a) the CuGB catalyst and (b) annealed catalyst. (c) C_2+_ products' partial current densities on the CuGB and annealed catalysts. (d) The ratio of faradaic efficiencies of ethylene and ethanol conversion on the CuGB and annealed catalysts.

We found that on the (100)-GB site, the C–O bond of *CH_2_CHO (ICOHP = −15.00 eV) is stronger than that on the Cu (100) site (ICOHP = −14.26 eV), which means the C–O bond is harder to break, thus inhibiting the ethylene formation. Our previous work found that the C–O bond length of *CH_2_CHO scales well with the difference of Δ*G*(C–O) and Δ*G*(+H)^46^ (Fig. 4a). The (100)-GB site is indeed located in the left part of the graph, which is the region where alcohol is generated.

Looking back at the bifurcation pathway, the stability of *CH_3_CHO and *O determines the direction of the reaction. The higher stability of *CH_3_CHO and lower stability of *O contribute to the ethanol formation; otherwise, the ethylene pathway is preferred. We found that the O of the optimized adsorbed *CH_3_CHO is closer to the undercoordinated Cu atom on (100)-GB (2.23 Å) than on (100)-ter (2.46 Å) (ESI Fig. S11†). This suggests a stronger interaction between *CH_3_CHO and the (100)-GB site, which is further validated by COHP analysis (ESI Fig. S12†). Meanwhile, we found that after the scission of the C–O bond of *CH_2_CHO, *O prefers to adsorb on the four-fold square site ((100)-ter) rather than the hexagonal site ((111) site) and disordered surface site ((100)-GB). This explains why the (100)-ter site can decrease Δ*G*(C–O) while (100)-GB has much higher Δ*G*(C–O).

Based on the above discussions, we figure out how GBs work to tune the selectivity for ethanol from theoretical insight. Furthermore, we propose our strategy to improve the selectivity for ethanol: introducing undercoordinated sites into the (100)-like square structure, through GB engineering, vacancy formation, and preparing high-index surfaces containing (100) steps or links like (310), and so on. Since the square site is conducive to C–C coupling, the adsorption strengths of *CH_3_CHO and acetaldehyde reduction intermediate *CH_3_CH_2_O are both enhanced on less coordinated sites (CN < 8) compared to planar (111) (CN = 9) and (100) (CN = 8) surfaces (Fig. 3b), which tune the reaction pathway to generate ethanol.

### Experimental validation

To verify the predicted trends of CO_2_R on GB-Cu, we synthesized a CuGB catalyst using a poly(vinylpyrrolidone) (PVP)-control electrodeposition method.^32^ Kanan *et al.* showed that the density of grain boundaries will decrease after annealing;^30^ we thus performed annealing at 200 °C on the synthesized CuGB catalyst as a contrast catalyst. Fig. 4a and b and ESI Fig. S14 and S15† show that with PVP as the additive, a high density of grain boundaries can be obtained while the particle sizes are not significantly changed. In contrast, after the annealing process, the CuGB surface will be much smoother, and the density of grain boundaries will significantly decrease. Our previous work had demonstrated that using the PVP-control method, the CuGB mainly consists of Cu^0^, therefore excluding the effect of the valence of Cu.^32^ We then compared the CO_2_R performance between these two catalysts using the H-cell system. As shown in Fig. 4c, the CuGB catalyst exhibits higher activity for C_2+_ products compared to the annealed sample at a wide potential window. Specifically, at −0.8 V *vs.* RHE, the CuGB catalyst yields 5.72 mA cm^−2^ current density for C_2+_ products, 6.9 times higher than that on annealed Cu. As for the selectivity, the annealed CuGB (Fig. 4d and ESI Fig. S16†) shows preference for ethylene production. We find that the ratio of ethylene to ethanol over the CuGB catalyst is lower than 1 at a relatively low overpotential (Fig. 4d and ESI Fig. S17†), which means that when introducing GBs on Cu, ethanol production is more favorable than that of ethylene. These experimental observations are perfectly consistent with our DFT predictions that CuGBs can promote C–C coupling to improve the activity for C_2+_ products, and at the same time, regulate the reaction pathway towards ethanol as well.

### Scaling up the CO_2_ reduction system

**Fig. 5:**
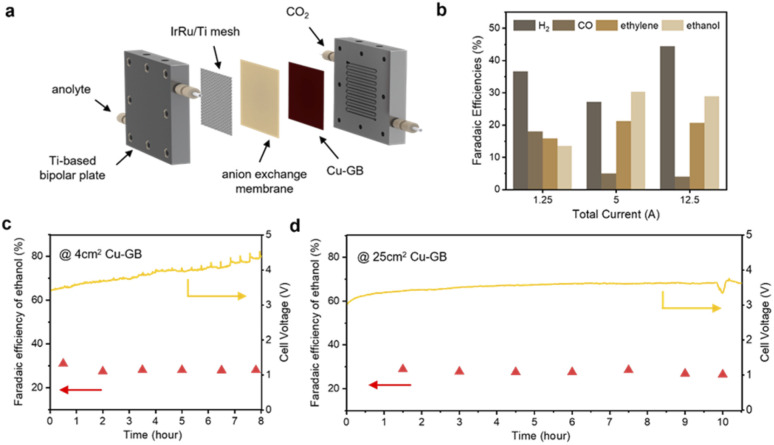
(a) Schematic of the MEA system. The gaskets are not shown. (b) Faradaic efficiencies of products in a 25 cm^2^ MEA system. (c) Stability test over a span of 8 h of CO_2_-electrolysis in a 4 cm^2^ MEA system at a total current of 1.0 A. (d) Stability test over a span of 10 h of CO_2_-electrolysis in a 25 cm^2^ MEA system at a total current of 12.5 A.

Based on the theoretical discussions, we suppose that CuGB catalysts can serve as promising candidates to catalyze CO_2_ reduction to ethanol. In order to explore the industrial application potential of the CuGB catalyst, we plan to build a large-area electrode and carry out performance testing at commercially relevant current density. Although many excellent efforts have been made and impressive ethanol selectivity achieved, the single-pass conversion for ethanol is still discouraging. A membrane electrode assembly (MEA) system can reduce the effect of mass transfer on the activity of the catalyst, and has been demonstrated to convert CO_2_ into desired products with high conversion rates.^47–49^ Therefore, we used the MEA system to verify the performance of the CuGB catalyst for ethanol production under practical conditions (Fig. 5a).

Details of the MEA system can be found in the Experimental procedures in the ESI† and our previous work.^49^ The electrode area in this work was enlarged to 25 cm^2^. In our 25 cm^2^ MEA system, the faradaic efficiency of ethanol production reached 26.8% at a total current of 12.5 A (Fig. 5b), still higher than that of ethylene (20.7%). Notably, in the reported studies with similar electrode areas (4–25 cm^2^), the corresponding optimal single-pass conversion of CO_2_ to ethanol reached a high level of 5.18% (Table S3†). Meanwhile, the single-pass conversion was not much compromised compared to other smaller electrode area reaction systems (∼1 cm^2^). It exhibited stable selectivity for C_2+_ products (>50%) and ethanol (∼28%) at 12.5 A total current over 10 h (Fig. 5d and ESI Fig. S18–S23†). Such high performance demonstrates the important role of GB sites in promoting ethanol production and validates the rationality of our calculation predictions. In the future, the catalyst deposition process needs to be continuously optimized to increase the GB density while further increasing the active area, which could lower the CO_2_ feed flow rate with improved conversion efficiency.

Although we achieved the tuning of the alcohols/ethylene ratio and the scaling up using the MEA reaction system, hydrogen is still the main product. This may be due to the electrodeposition method, where a certain exposure of the substrate exists in the obtained electrodes. In the future, the wetting state of the electrode needs to be tuned to match the electrodeposition process and to construct a three-phase interface.^7^ Also, increasing CO_2_ pressure can be considered.^50^

## Conclusions

In summary, the origin of enhanced catalytic activity for CO_2_ electrochemical reduction to C_2+_ products, especially ethanol, over Cu GBs was illustrated. GBs introduced to Cu (111) and Cu (100) surfaces have significant impacts on the adsorption behaviors of key intermediates, which prompts CO_2_ activation, CO protonation and CO–CHO coupling. Moreover, the low coordinated sites on the GBs grant the selectivity for ethanol by stabilizing acetaldehyde and weakening *O adsorption. These trends are validated by experimental results. Inspired by the unique roles of GBs, we propose a universal strategy for improving ethanol generation, which is introducing undercoordinated sites into (100)-like square structures. Furthermore, since Cu GBs show exciting potential to catalyze CO_2_ to ethanol, we thus scaled up the CO_2_ reduction system using a membrane electrode assembly system and obtained an overall 12.5 A current and a single pass conversion of 5.18% for ethanol over the synthesized Cu-GB catalyst.

## Data availability

The data that support the findings of this study are available within the article and its ESI,† or from the corresponding author on reasonable request.

## Author contributions

Dongfang Cheng: methodology; investigation; visualization; formal analysis; validation; writing – original draft. Gong Zhang: methodology; investigation; formal analysis; writing – original draft. Lulu Li: investigation; formal analysis. Xiangcheng Shi: investigation; formal analysis. Shiyu Zhen: investigation; formal analysis. Zhi-Jian Zhao: supervision; writing – review & editing; resources and funding acquisition. Jinlong Gong: supervision; writing – review & editing; resources and funding acquisition.

## Conflicts of interest

There are no conflicts to declare.

## Supplementary Material

SC-014-D3SC02647G-s001

## References

[cit1] Garcia de Arquer F. P., Dinh C. T., Ozden A., Wicks J., McCallum C., Kirmani A. R., Nam D. H., Gabardo C., Seifitokaldani A., Wang X., Li Y. C., Li F., Edwards J., Richter L. J., Thorpe S. J., Sinton D., Sargent E. H. (2020). Science.

[cit2] Gao D., Li W., Wang H., Wang G., Cai R. (2022). Trans. Tianjin Univ..

[cit3] Yao D., Tang C., Wang P., Cheng H., Jin H., Ding L.-X., Qiao S.-Z. (2022). Chem. Eng. Sci..

[cit4] Shao B., Zhang Y., Sun Z., Li J., Gao Z., Xie Z., Hu J., Liu H. (2022). Green Chem. Eng..

[cit5] Zhang L., Hu S., Zhu X., Yang W. (2017). J. Energy Chem..

[cit6] Shi R., Wang Z., Zhao Y., Waterhouse G. I. N., Li Z., Zhang B., Sun Z., Xia C., Wang H., Zhang T. (2021). Nat. Catal..

[cit7] Shi R., Guo J., Zhang X., Waterhouse G. I. N., Han Z., Zhao Y., Shang L., Zhou C., Jiang L., Zhang T. (2020). Nat. Commun..

[cit8] Yao S., Wei Y., Lu Z., Guo S., Chen J., Yu Z. (2023). Renewables.

[cit9] Nitopi S., Bertheussen E., Scott S. B., Liu X., Engstfeld A. K., Horch S., Seger B., Stephens I. E. L., Chan K., Hahn C., Norskov J. K., Jaramillo T. F., Chorkendorff I. (2019). Chem. Rev..

[cit10] Dinh C.-T., Burdyny T., Kibria M. G., Seifitokaldani A., Gabardo C. M., García de Arquer F. P., Kiani A., Edwards J. P., De Luna P., Bushuyev O. S., Zou C., Quintero-Bermudez R., Pang Y., Sinton D., Sargent E. H. (2018). Science.

[cit11] Yuan X., Chen S., Cheng D., Li L., Zhu W., Zhong D., Zhao Z. J., Li J., Wang T., Gong J. (2021). Angew. Chem., Int. Ed..

[cit12] Cheng D., Wei Z., Zhang Z., Broekmann P., Alexandrova A. N., Sautet P. (2023). Angew. Chem., Int. Ed..

[cit13] Yang Y., Louisia S., Yu S., Jin J., Roh I., Chen C., Fonseca Guzman M. V., Feijoo J., Chen P. C., Wang H., Pollock C. J., Huang X., Shao Y. T., Wang C., Muller D. A., Abruna H. D., Yang P. (2023). Nature.

[cit14] Xue Y., Guo Y., Cui H., Zhou Z. (2021). Small Methods.

[cit15] Feng X., Jiang K., Fan S., Kanan M. W. (2015). J. Am. Chem. Soc..

[cit16] Li C. W., Ciston J., Kanan M. W. (2014). Nature.

[cit17] Zhong D., Zhao Z. J., Zhao Q., Cheng D., Liu B., Zhang G., Deng W., Dong H., Zhang L., Li J., Li J., Gong J. (2021). Angew. Chem., Int. Ed..

[cit18] Hahn C., Hatsukade T., Kim Y. G., Vailionis A., Baricuatro J. H., Higgins D. C., Nitopi S. A., Soriaga M. P., Jaramillo T. F. (2017). Proc. Natl. Acad. Sci. U. S. A..

[cit19] Deng B., Zhao X., Li Y., Huang M., Zhang S., Dong F. (2022). Sci. China: Chem..

[cit20] Zhuang T.-T., Liang Z.-Q., Seifitokaldani A., Li Y., De Luna P., Burdyny T., Che F., Meng F., Min Y., Quintero-Bermudez R., Dinh C. T., Pang Y., Zhong M., Zhang B., Li J., Chen P.-N., Zheng X.-L., Liang H., Ge W.-N., Ye B.-J., Sinton D., Yu S.-H., Sargent E. H. (2018). Nat. Catal..

[cit21] Ma W., Xie S., Liu T., Fan Q., Ye J., Sun F., Jiang Z., Zhang Q., Cheng J., Wang Y. (2020). Nat. Catal..

[cit22] Clark E. L., Hahn C., Jaramillo T. F., Bell A. T. (2017). J. Am. Chem. Soc..

[cit23] Qiu N., Li J., Wang H., Zhang Z. (2022). Sci. China Mater..

[cit24] Li Y. C., Wang Z., Yuan T., Nam D. H., Luo M., Wicks J., Chen B., Li J., Li F., de Arquer F. P. G., Wang Y., Dinh C. T., Voznyy O., Sinton D., Sargent E. H. (2019). J. Am. Chem. Soc..

[cit25] Wang X., Wang Z., García de Arquer F. P., Dinh C.-T., Ozden A., Li Y. C., Nam D.-H., Li J., Liu Y.-S., Wicks J., Chen Z., Chi M., Chen B., Wang Y., Tam J., Howe J. Y., Proppe A., Todorović P., Li F., Zhuang T.-T., Gabardo C. M., Kirmani A. R., McCallum C., Hung S.-F., Lum Y., Luo M., Min Y., Xu A., O'Brien C. P., Stephen B., Sun B., Ip A. H., Richter L. J., Kelley S. O., Sinton D., Sargent E. H. (2020). Nat.
Energy.

[cit26] Luo M., Wang Z., Li Y. C., Li J., Li F., Lum Y., Nam D. H., Chen B., Wicks J., Xu A., Zhuang T., Leow W. R., Wang X., Dinh C. T., Wang Y., Wang Y., Sinton D., Sargent E. H. (2019). Nat. Commun..

[cit27] Li F., Li Y. C., Wang Z., Li J., Nam D.-H., Lum Y., Luo M., Wang X., Ozden A., Hung S.-F., Chen B., Wang Y., Wicks J., Xu Y., Li Y., Gabardo C. M., Dinh C.-T., Wang Y., Zhuang T.-T., Sinton D., Sargent E. H. (2019). Nat. Catal..

[cit28] Zhou Y., Che F., Liu M., Zou C., Liang Z., De Luna P., Yuan H., Li J., Wang Z., Xie H., Li H., Chen P., Bladt E., Quintero-Bermudez R., Sham T. K., Bals S., Hofkens J., Sinton D., Chen G., Sargent E. H. (2018). Nat. Chem..

[cit29] Hoang T. T. H., Verma S., Ma S., Fister T. T., Timoshenko J., Frenkel A. I., Kenis P. J. A., Gewirth A. A. (2018). J. Am. Chem. Soc..

[cit30] Verdaguer-Casadevall A., Li C. W., Johansson T. P., Scott S. B., McKeown J. T., Kumar M., Stephens I. E., Kanan M. W., Chorkendorff I. (2015). J. Am. Chem. Soc..

[cit31] Feng X., Jiang K., Fan S., Kanan M. W. (2016). ACS Cent. Sci..

[cit32] Chen Z., Wang T., Liu B., Cheng D., Hu C., Zhang G., Zhu W., Wang H., Zhao Z. J., Gong J. (2020). J. Am. Chem. Soc..

[cit33] Dong C., Fu J., Liu H., Ling T., Yang J., Qiao S. Z., Du X.-W. (2017). J. Mater. Chem. A.

[cit34] Shi C., Hansen H. A., Lausche A. C., Norskov J. K. (2014). Phys. Chem. Chem. Phys..

[cit35] Peterson A. A., Nørskov J. K. (2012). J. Phys. Chem. Lett..

[cit36] Sandberg R. B., Montoya J. H., Chan K., Nørskov J. K. (2016). Surf. Sci..

[cit37] Calle-Vallejo F., Tymoczko J., Colic V., Vu Q. H., Pohl M. D., Morgenstern K., Loffreda D., Sautet P., Schuhmann W., Bandarenka A. S. (2015). Science.

[cit38] Bondue C. J., Calle-Vallejo F., Figueiredo M. C., Koper M. T. M. (2019). Nat. Catal..

[cit39] Liu S. G., Huang S. P. (2019). J. Catal..

[cit40] Ma X., Xin H. (2017). Phys. Rev. Lett..

[cit41] Kim K.-S., Kim W. J., Lim H.-K., Lee E. K., Kim H. (2016). ACS Catal..

[cit42] Liu X., Schlexer P., Xiao J., Ji Y., Wang L., Sandberg R. B., Tang M., Brown K. S., Peng H., Ringe S., Hahn C., Jaramillo T. F., Norskov J. K., Chan K. (2019). Nat. Commun..

[cit43] Goodpaster J. D., Bell A. T., Head-Gordon M. (2016). J. Phys. Chem. Lett..

[cit44] Calle-Vallejo F., Koper M. T. (2013). Angew. Chem., Int. Ed..

[cit45] Hanselman S., Koper M. T. M., Calle-Vallejo F. (2018). ACS Energy Lett..

[cit46] Cheng D., Zhao Z. J., Zhang G., Yang P., Li L., Gao H., Liu S., Chang X., Chen S., Wang T., Ozin G. A., Liu Z., Gong J. (2021). Nat. Commun..

[cit47] Miao R. K., Xu Y., Ozden A., Robb A., O'Brien C. P., Gabardo C. M., Lee G., Edwards J. P., Huang J. E., Fan M., Wang X., Liu S., Yan Y., Sargent E. H., Sinton D. (2021). Joule.

[cit48] Ripatti D. S., Veltman T. R., Kanan M. W. (2019). Joule.

[cit49] Zhang G., Zhao Z. J., Cheng D., Li H., Yu J., Wang Q., Gao H., Guo J., Wang H., Ozin G. A., Wang T., Gong J. (2021). Nat. Commun..

[cit50] Jin J., Wicks J., Min Q., Li J., Hu Y., Ma J., Wang Y., Jiang Z., Xu Y., Lu R., Si G., Papangelakis P., Shakouri M., Xiao Q., Ou P., Wang X., Chen Z., Zhang W., Yu K., Song J., Jiang X., Qiu P., Lou Y., Wu D., Mao Y., Ozden A., Wang C., Xia B. Y., Hu X., Dravid V. P., Yiu Y. M., Sham T. K., Wang Z., Sinton D., Mai L., Sargent E. H., Pang Y. (2023). Nature.

