# Supplementary material for: Guiding catalytic CO2 reduction to ethanol with copper grain boundaries
Source: Chem Sci. 2023 Jun 22;14(29):7966–72. doi: 10.1039/d3sc02647g (PMC10370575; doi:10.1039/d3sc02647g)
Supplement: SC-014-D3SC02647G-s001 [file SC-014-D3SC02647G-s001.pdf]

Electronic Supplementary Material (ESI) for Chemical Science.
This journal is © The Royal Society of Chemistry 2023

Supporting Information

Guiding Catalytic CO_2 Reduction toward Ethanol with Copper Grain Boundaries

Dongfang Cheng[1,2#], Gong Zhang,[1,2#], Lulu Li[1,2], Xiangcheng Shi[1,2,3], Shiyu Zhen[1,2], Zhi-Jian Zhao* [1,2,3,4] and Jinlong Gong* [1,2,3,4]

[1]*Key Laboratory for Green Chemical Technology of Ministry of Education, School of Chemical Engineering & Technology, Tianjin University, Tianjin 300072, China*

[2]*Collaborative Innovation Center for Chemical Science & Engineering (Tianjin), Tianjin 300072, China*

[3]*Joint School of National University of Singapore and Tianjin University, International Campus of Tianjin University, Binhai New City, Fuzhou 350207, China*

[4]*Haihe Laboratory of Sustainable Chemical Transformations, Tianjin 300192, China*

# These authors contributed equally to this work.

* E-mail: zjzhao@tju.edu.cn, jlgong@tju.edu.cn

Computational Details

The types of grain boundaries are diverse. Considering (111) and (100) facets occupy most of the Cu surface, as well as the computational cost, we constructed two representative Cu GBs, Cu∑3/(111) GB and Cu∑5/(100) GB. These two models were built according to coincidence site lattice theory[1]. The modeling process of Cu∑3/(111) GB was described in detail in our previous work[2]. Cu∑5/(100) GB was built by exposing the (100) plane, the rotation angle is 53.1°. The atomic coordinates of the two GBs are attached at the end. We acknowledge that there are numerous types of grain boundaries. Grain boundary is known as one kind of defect which contains low-coordinated sites and strain effect. We expect these two representative models, Cu∑3/(111) and Cu∑5/(100), can capture the key features of grain boundaries.

All calculations were performed by DFT as implemented in Vienna ab initio simulation package[3]. BEEF-vdW exchange correlation functional was used[4]. The cut off energy is 400 eV. The interactions between the atomic cores and electrons were described by the projector augmented wave method[5]. All structures were optimized until the force on each atom has been less than 0.02 eV/Å. 3×1×1 and 1×3×1 k-point Monkhorst–Pack mesh sampling was employed for the Cu∑3/(111) GB and Cu∑5/(100) GB models respectively. The transition state search was conducted with climbing image nudged elastic band (CI-NEB) method, followed by the dimer method to converge the saddle point within 0.05 eV/Å. The bottom two layers are fixed while the top layer can relax with adsorbate when performing structure optimization. The free energy for intermediate was calculated as $\Delta G = \Delta E + \Delta ZPE - T\Delta S$, where ΔE is the reaction energy change from DFT calculations, ΔZPE is the change of zero point energies (ZPE) which is calculated with the vibrational frequencies of absorbates and molecules, and ΔS is the entropy change in the reaction. T is temperature and is set to 298 K. The computational hydrogen electrode (CHE) model[6] was employed to determine free energies of intermediates. Solvation corrections are conducted according to Calle-Vallejo's work[7]. When considering CO-CO coupling step, one layer charged water is established to represent electrochemical interface[8].

The COHP analysis are performed by LOBSTER[9].

Experimental Procedures

Chemicals and Materials

Commercially available carbon-based gas diffusion layers (GDLs, AvCarb GDS3250) were purchased from Xima Laya Photo-Electric Technology Co., Ltd., China. poly(vinylpyrrolidone) (PVP-K30) was purchased from Solarbio Science & Technology Co. Ltd. KOH (95%), K_2CO_3 (99.99%) and $Cu(NO)_3 \cdot 3H_2O$ (99.99%) were purchased from Sigma-Aladdin. Nitric acid (HPLC) was purchased from Tianjin Kemiou Chemical Reagent Co., Ltd. The reagents were used without any purification process. Deionized water (18.25 MΩ·cm) supplied by a Millipore Direct-Q5 System was used in the whole experimental process. CO_2, N_2, Ar, H_2 and O_2 were all supplied by Air Liquide (≥99.999%).

Preparation of GB-Cu electrodes

The GB-Cu catalyst was in situ grown on carbon paper by additive-controlled electrodeposition. In a typical synthesis, 6.5 mmol of $Cu(NO_3)_2$, with 0.5 g PVP, was dissolved in 50 mL ultrapure water with stirring for 30 min until the solution turns into a transparent one. Next, carbon paper, cut into 1.5 cm × 1.5 cm for each piece, was used as the cathode. GB-Cu was in situ electrodeposited at -0.3 V (vs. Ag/AgCl) on the GDL for 1800 seconds. After electrodeposition, the GDL was rinsed by isopropanol, following by the deionized water for several times to remove residual PVP and Cu^{2+} ions on the surface and then dried by the nitrogen gas.

Preparation of annealed GB-Cu electrodes

The annealed GB-Cu electrode was obtained by annealing GB-Cu electrode at 200°C for two hours under N_2 atmosphere.

Characterizations.

Transmission Electron Microscopy (TEM), High-resolution TEM (HRTEM) images were obtained at 200 kV (JEOL JEM-2100F).

Electrochemical reduction of CO_2 in a H-type cell

All electrochemical measurements were conducted in a custom gas-tight H-type electrochemical cell machined from PMMA. (manufactured by Gaossunion Co., Ltd.). The cell was sonicated in 20 wt. % nitric acid and thoroughly rinsed with the deionized water prior to all experimentation. The working and counter electrodes were parallel and separated by an anion-conducting membrane (FAA-3-50, FuMA-Tec). Gas dispersion frits were incorporated into both electrode chambers in order to provide ample electrolyte-gas mixing. The exposed geometric surface area of each electrode was 1 cm^2 and the electrolyte volume of each electrode chamber was 10 mL. The counter electrode was a glassy carbon plate that was also sonicated in 20 wt. % nitric acid prior to all experimentation. The working electrode potential was referenced against a Ag/AgCl electrode (saturated KCl electrolyte). A 0.05 M K_2CO_3 solution was used as the electrolyte. Metallic impurities in the as-prepared electrolyte were removed before electrolysis by chelating the solution with Chelex 100 (Na form, purchased from Sigma-Aladdin). Both electrode chambers were sparged with CO_2 at a rate of 5 sccm for 30 min prior to and throughout the duration of all electrochemical measurements. Upon saturation with CO_2 the pH of the electrolyte was 6.8. Electrochemistry was performed using a Autolab PGSTAT204 potentiostat. All electrochemical measurements were recorded versus the reference electrode and converted to the RHE scale. The electrocatalytic activity of each sample was assessed by conducting chronoamperometry for 70 min. Each electrode was tested at least three times in order to ensure the statistical relevance of the observed trends.

Electrochemical reduction of CO_2 in the MEA system

The MEA cell (manufactured by Gaossunion Co., Ltd.) consists of a titanium a cathode bipolar plate with serpentine flow field, an anode bipolar plate with parallel flow field, associated nuts, bolts and insulating kit. The geometric area of each flow field is 4 and 25cm^2. An AEM membrane (FAA-3-30, Fumatech) was activated in 0.1 M KOH for 24 hours, washed with the deionized water prior to use. The anode consisted of a IrRu alloy deposited on a 200 mesh Ni grid. A direct current power supply (UTP1300, UNI-T Group Co., Ltd) was used to apply current to the MEA. A Corrtest CS350M in a galvanostatic mode was used to measure the cell voltage. No iR compensation was applied. Aqueous KOH electrolyte (10 mM0.1 M) was used as the anolyte and was circulated using a peristaltic pump (EC200-01, Gaossunion Co., Ltd.). The electrolyte flow rate was kept at 20 mL min^{-1}. The flow rate of the CO_2 gas flowing into the cathode flow field was kept at 50 sccm and 600 sccm by a mass flow controller (MC-Series, Alicat Scientific) for 4cm^2 and 25cm^2, respectively. CO_2 was flowed through a homemade humidifier (7/8 full of Milli-Q water, room temperature) prior to the MEA. The flow rate of the CO_2 gas flowing out the cathode flow field was also measured by a flowmeter (M-Series, Alicat Scientific). The liquid products carried by CO_2 gas are absorbed by low-temperature ultra-purity water obtained from an ice salt bath.

Analysis of CO_2 reduction products

During electrolysis, gas products were quantified using an on-line gas chromatography system (GC7890B, Agilent Technologies, Inc.). The thermal conductivity detector (TCD) connected to a MolSieve 5A packed column (Agilent Technologies, Inc.) to detect H_2, O_2 and N_2 and a back flame ionization detector (FID) connected to a Porapak Q packed column (Agilent Technologies, Inc.) to detect CO. A methanizer was installed to enable the back FID to detect CO with 1000 times higher sensitivity. A front FID connected to an HP-PLOT Al_2O_3 capillary column (Agilent Technologies, Inc.) to detect hydrocarbons (C1~C3). Ar was used as the carrier gas. After passing through the reactor, the gas was allowed to flow directly into the gas sampling loop of the gas chromatography for online gaseous product analysis.

In the performance test using H-type cell and the MEA system, the liquid products were collected from the cathode and anode chambers. The liquid products were analyzed by headspace gas chromatography (HS-GC) and 1H-NMR. HS-GC measurements were carried out using a BCHP HS-2 Headspace Sampler with GC2060 gas chromatography (Shanghai Ruimin Instrument Co., Ltd.). Typically, 10 mL vials were filled with 3 mL of the liquid sample and sealed. They were heated to 70 ◦C over 20 min in the headspace sampler and 1mL of the headspace gas composition was automatically injected into the GC. The sample loop (110 ◦C) and transfer line (110 ◦C) were both heated to avoid condensation. Ar was used as the carrier gas. An HP-INNOWax capillary column (Length: 60 m; ID: 0.32 mm; Film: 0.5 μm, Agilent) was used to separate the compounds in the sample. 1H-NMR was performed using AVANCE IIITM HD 400 MHz NanoBAY. The water suppression method was used. DMSO (10 mM) and phenol (50 mM) were added as internal standards. For CO2 reduction products showing multiple sets of NMR peaks, the set of peaks with the highest intensity were chosen for calibration and quantification.

Supplementary Fig. S1. (a) The linear relationship between adsorption energy of *COOH, *CHO and *CO. (b) Reaction free energy of *CO protonation to *CHO and *COH, and *CO release to gaseous CO at -0.5V (vs. RHE).

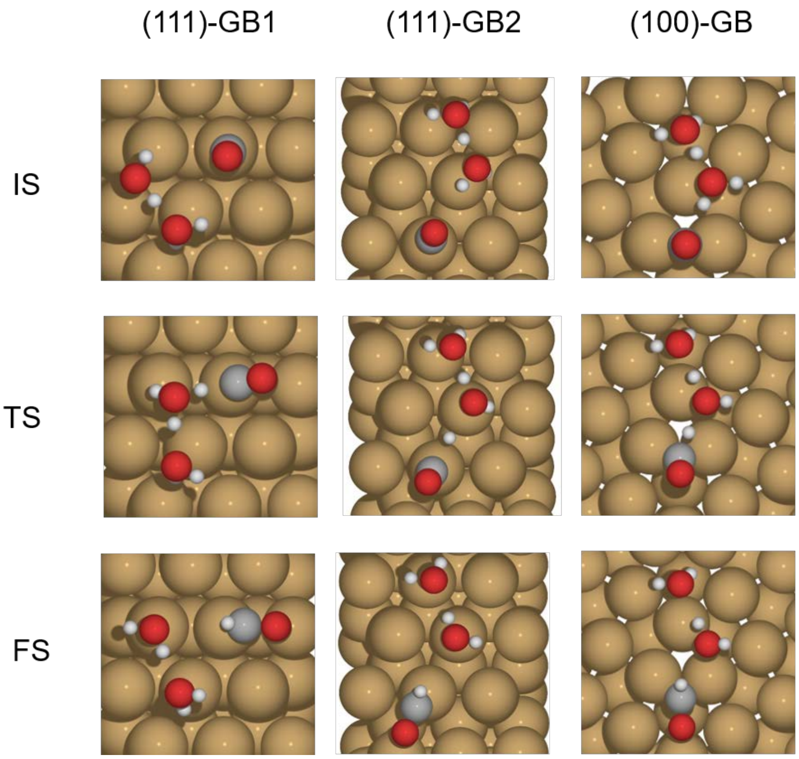

Supplementary Fig. S2. Configurations of IS, TS and FS for *CO protonation on GBs.

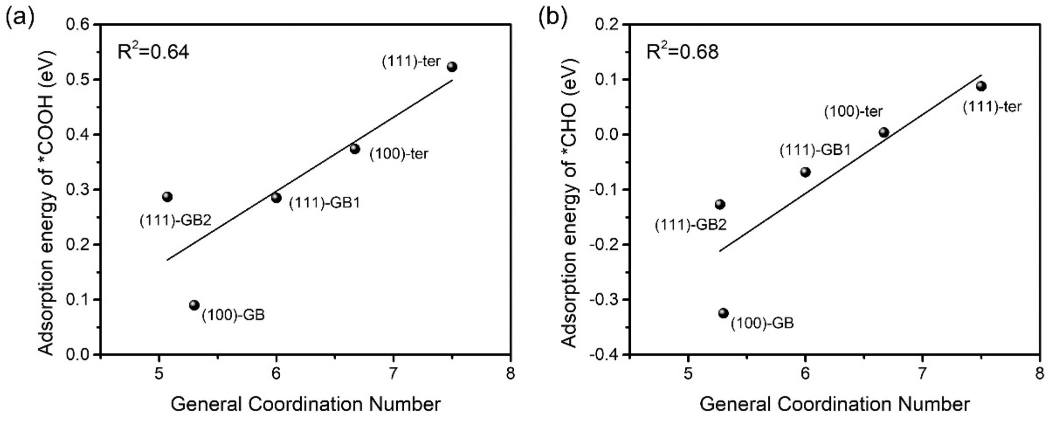

Supplementary Fig. S3. The correlation of general coordination number with the adsorption energy of *COOH and *CHO.

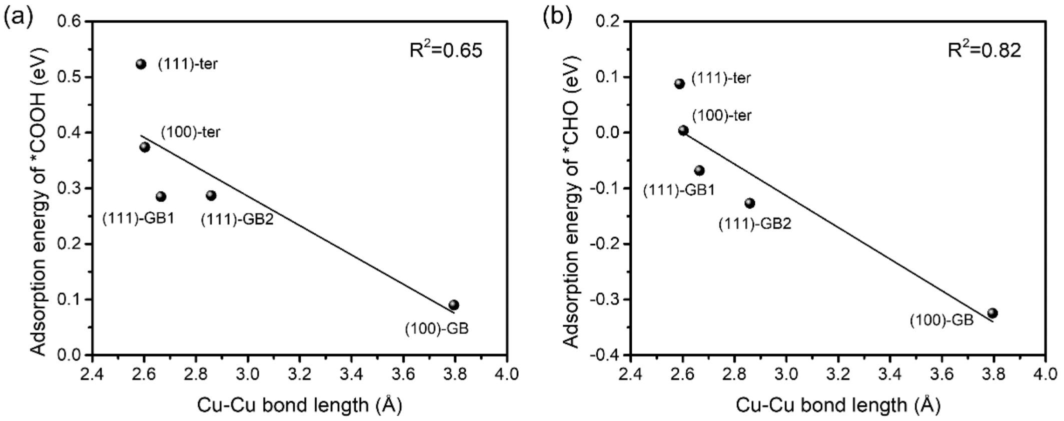

Supplementary Fig. S4. The correlation of Cu-Cu bond length with the adsorption energy of *COOH and *CHO.

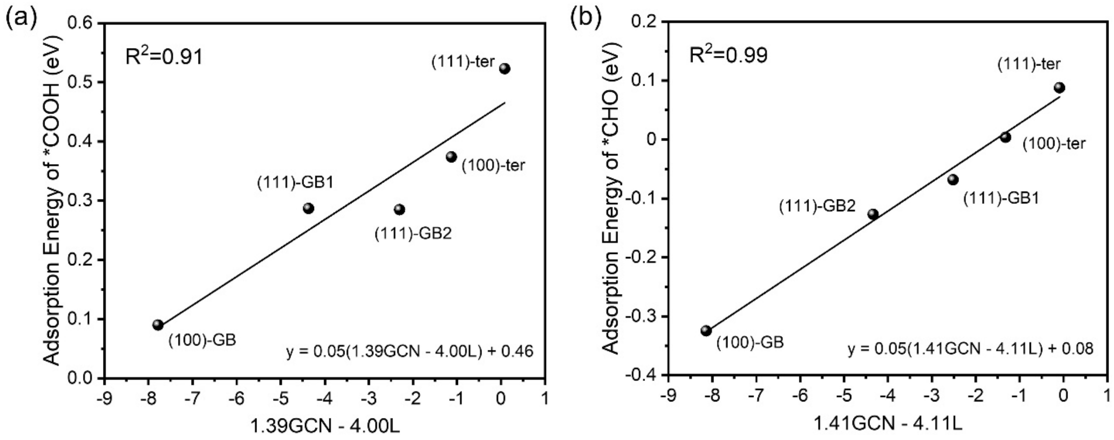

Supplementary Fig. S5. Relationships between the adsorption energy of (a) *COOH (b) *CHO and the description combined general coordination number(GCN) and Cu-Cu bond length(L).

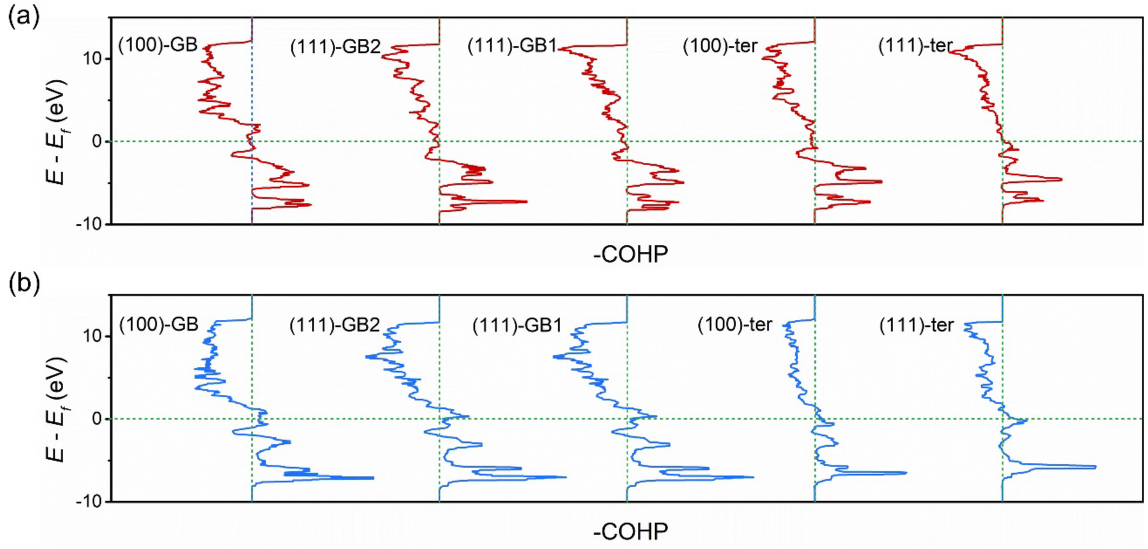

Supplementary Fig. S6. The crystal orbital Hamilton population(COHP) between the (a) *COOH, (b)*CHO and surface Cu.

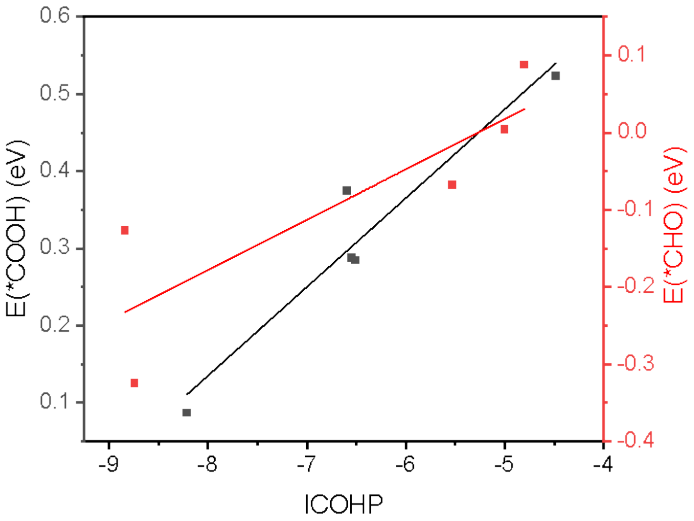

Supplementary Fig. S7. The correlation of ICOHP with the adsorption energy of *COOH and *CHO.

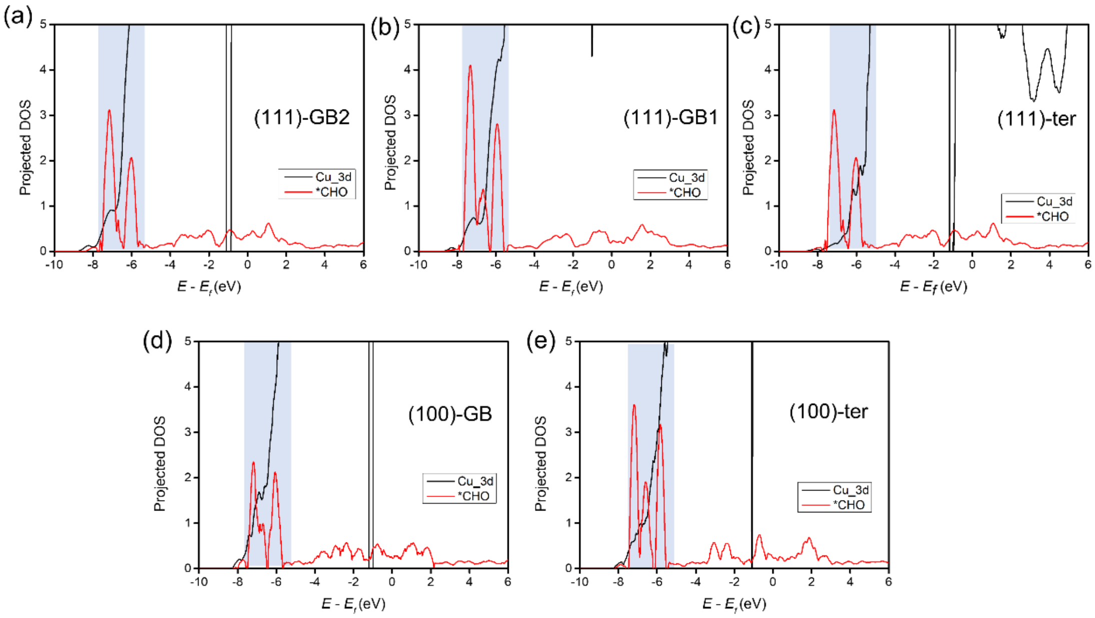

Supplementary Fig. S8. PDOS of Cu d-orbital and adsorbed *CHO on (a) (111)-GB2, (b) (111)-GB1, (c) (111)-ter, (d) (100)-GB and (e) (100)-ter.

Supplementary Fig. S9. Free energy diagrams of bifurcation pathways for (a) ∑3 /(111) GBs and (b) ∑5 /(100) GBs at zero electrode potential.

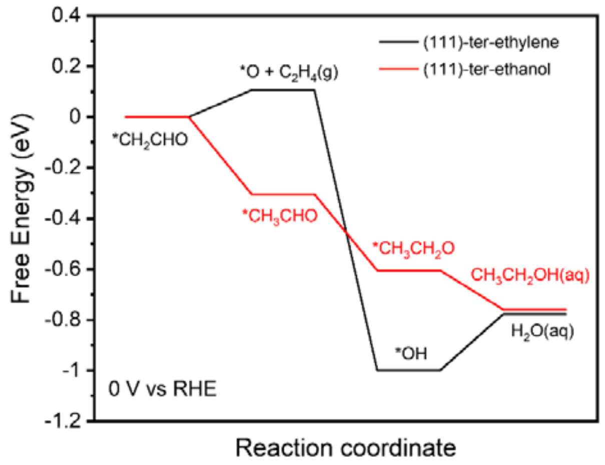

Supplementary Fig. S10. Free energy diagrams of bifurcation pathway on (111)-ter site at zero electrode potential.

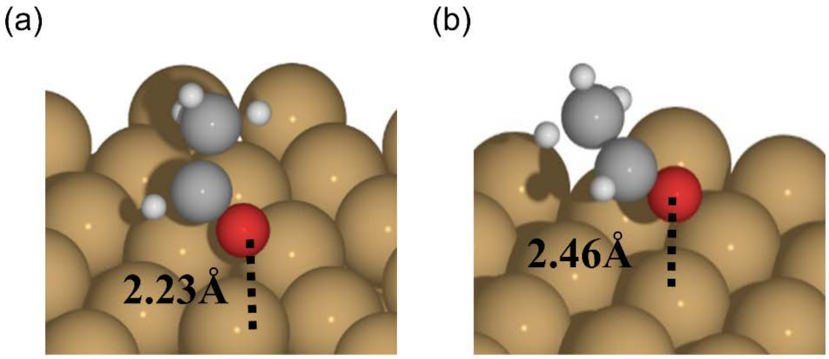

Supplementary Fig. S11. Schematic illustration of intermediate *CH_3CHO intermediate on (a) (100)-GB and (b) (100)-ter.

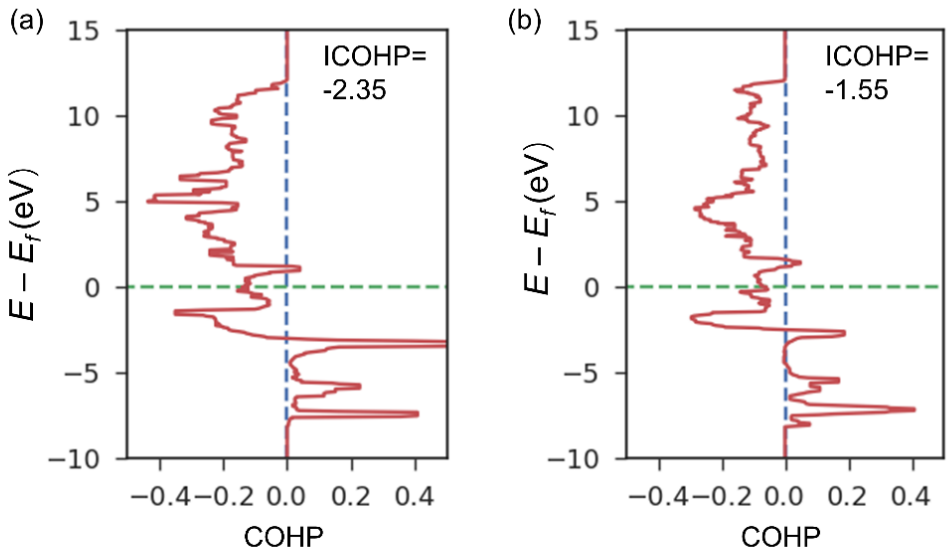

Supplementary Fig. S12. The crystal orbital Hamilton population (COHP) between the O of *CH_3CHO and (a) (100)-GB, (b) (100)-ter.

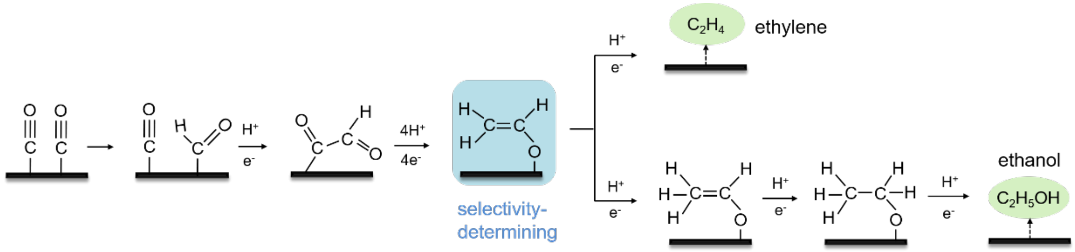

Supplementary Fig. S13. Proposed mechanism for CO_2R to C2 products on Cu GBs.

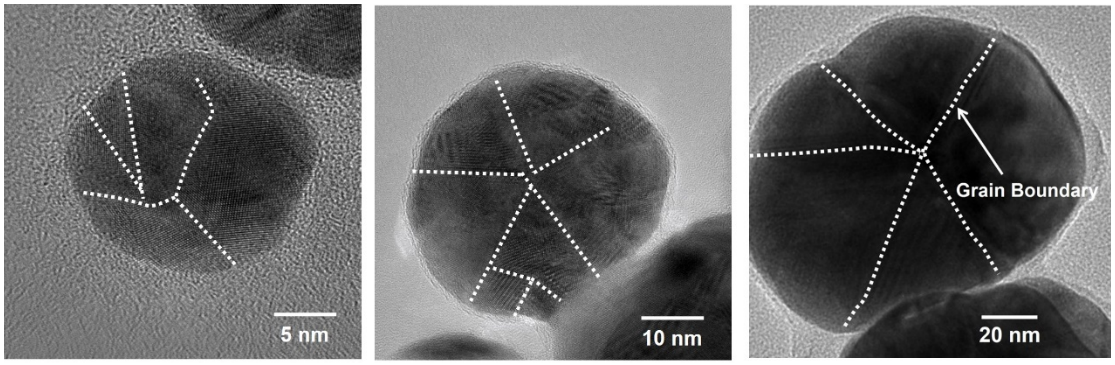

Supplementary Fig. S14. HRTEM images of CuGB catalysts.

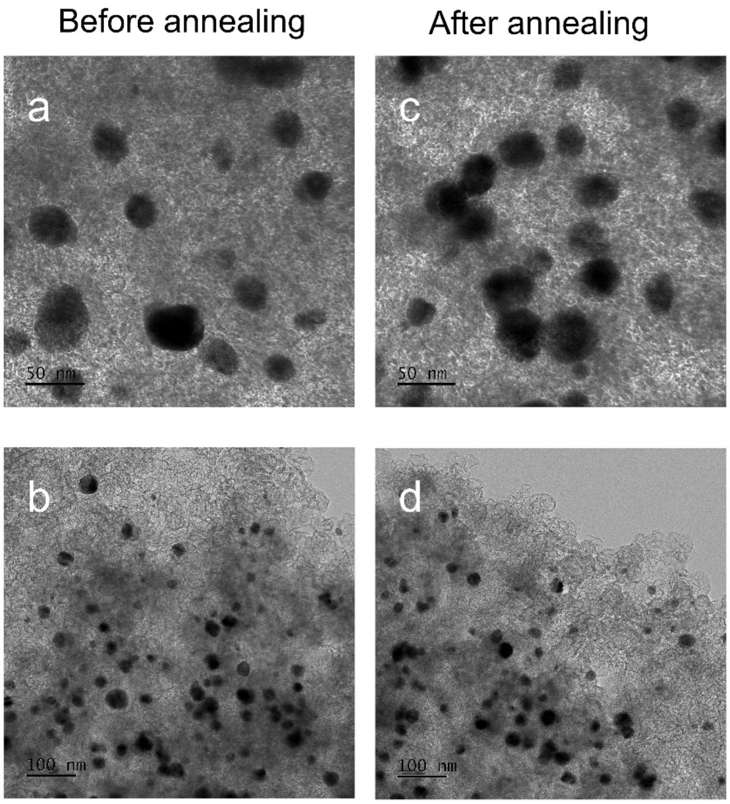

Supplementary Fig. S15. TEM images of CuGB catalysts (a)(b)before annealing.(c)(d)after annealing.

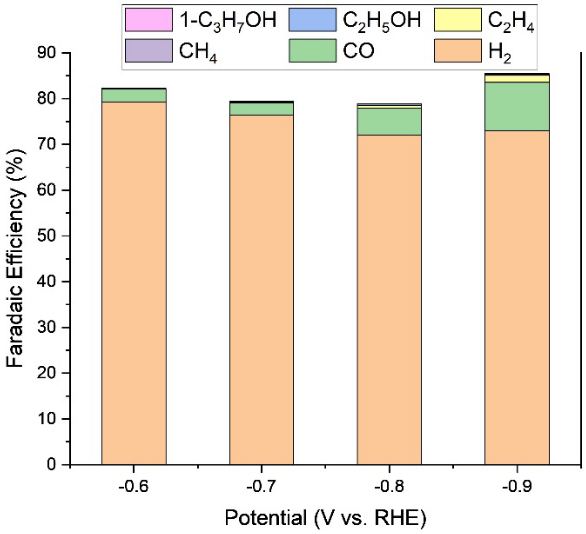

Supplementary Fig. S16. Faradaic efficiencies of reduction products on the annealed Cu-GB sample as a function of different current densities in H-cell system.

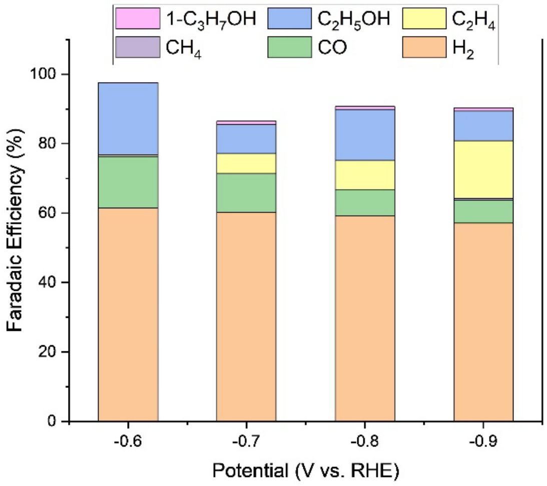

Supplementary Fig. S17. Faradaic efficiencies of reduction products on the Cu-GB sample as a function of different current densities in H-cell system.

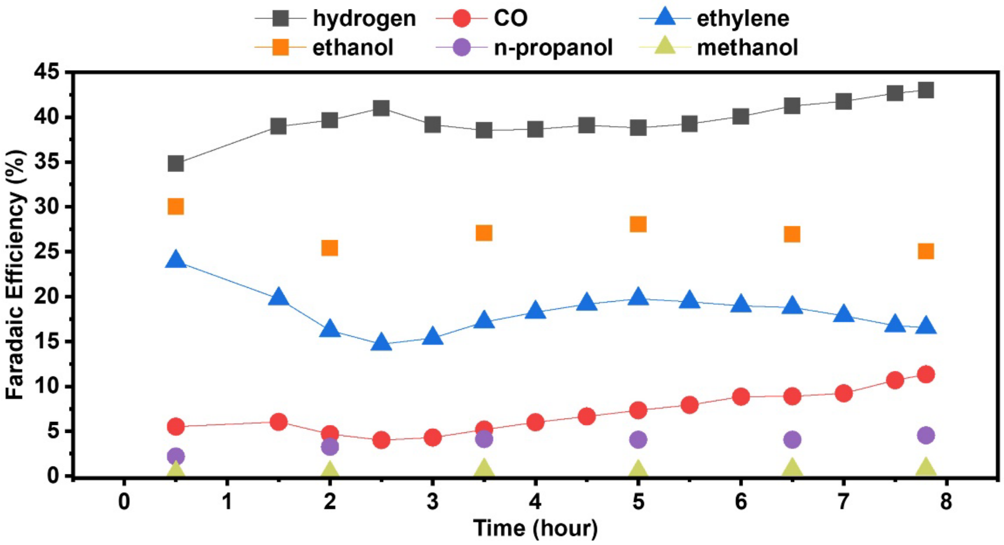

Supplementary Fig. S18. Stability test over a span of 8 h of CO_2-electrolysis in a 4 cm^2-MEA system at the total current of 1.0 A for all products.

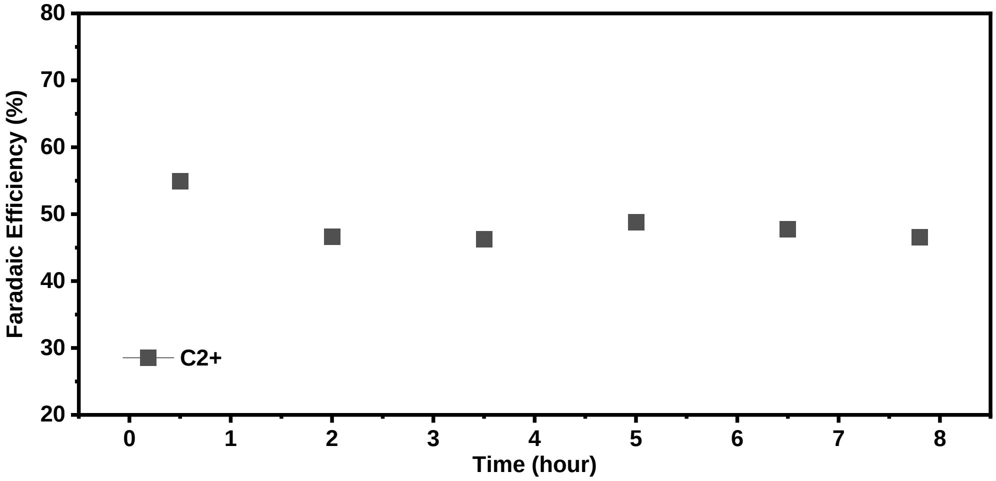

Supplementary Fig. S19. Stability test over a span of 8 h of CO_2-electrolysis in a 4 cm^2-MEA system at the total current of 1.0 A for C2+ products (including ethylene, ethanol, and n-propanol).

Supplementary Fig. S20. Faradaic efficiencies of reduction products on the Cu-GB sample as a function of different current densities in 25 cm^2-MEA system.

Supplementary Fig. S21. Current density as a function of cell voltage in 25 cm^2-MEA system.

Supplementary Fig. S22. Faradaic efficiencies of all reduction products on the Cu-GB in 25 cm^2-MEA system during the stability test.

Supplementary Fig. S23. Faradaic efficiencies of C2+ products (including ethylene, ethanol, and n-propanol) on the Cu-GB in 25 cm^2-MEA system during the stability test.

Table S1. Adsorption energies of *COOH, *CHO and *CO on different sites. All values are in eV.

	*COOH	*CHO	*CO
(111)-GB1	0.28	-0.07	-0.67
(111)-GB2	0.29	-0.13	-0.58
(111)-ter	0.52	0.09	-0.49
(100)-GB	0.08	-0.32	-0.74
(100)-ter	0.37	0.00	-0.64

Table S2. Adsorption energies of $*CH_3CHO$ and $*CH_3CH_2O$ on different sites. All values are in eV.

	$*CH_3CHO$	$*CH_3CH_2O$
Cu (111)	0.08	-0.59
Cu (100)	-0.22	-1.01
Cu (211)	-0.42	-1.18
Cu (211)k	-0.46	-1.14
3AD@Cu (111)	-0.50	-1.25

Table S3. Summary of total current and conversion efficiency towards ethanol from different system.

**Catalyst**	***I*(EtOH)(mA)**	**Single-pass conversion (CO_2-EtOH)(%)**	**Reference**
Cu-GB-4cm^2	300	2.32	this work
Cu-GB-25cm^2	3350	5.18	this work
Cu/Ag	102.5	0.48	***J. Am. Chem. Soc.*** 141, 8584−8591(2019)
Ce(OH)x/Cu	128	0.59	***Nat. Commun.*** 10**,** 5814 (2019)
Cu_2S-Cu	100	0.46	***Nat. Catal.*** 1, 421–428 (2018)
Boron-doped Cu	18.9	0.09	***Nat. Chem.*** 10, 974–980 (2018)
CuO/ZnO	82	0.95	***Angew.Chem.*** 58,15036 –15040(2019)
N-C/Cu	156	0.72	***Nat. Energy*** 5, 478–486 (2020)
FeTPP[Cl]/Cu	124	0.58	***Nat. Catal.*** 3, 75–82 (2020)
Cu	287.5	0.83	***Joule*** 5, 2742–2753 (2021)
Cu	195.5	0.57	***Joule*** 3, 2777–2791(2019)
Cu_2O/Cu	111	1.28	***Nat. Commun.*** 14, 501 (2023)
Hierarchical Cu	80	0.93	***J. Am. Chem. Soc***. 143, 8011–8021(2021)
CuAg	75	2.49	***J. Am. Chem. Soc***. 140, 5791–5797(2018)
F-Cu	240	2.79	***Nat. Catal.*** 3, 478–487 (2020)

Table S4. Cartesian Coordination of the Cu∑3/(111) GB
Unit cell:

	x /Å	y /Å	z /Å
x	7.77	0.00	0.00
y	0.00	23.51	0.00
z	0.00	0.00	24.21

Atom	x /Å	y /Å	z /Å
Cu	6.57	15.16	8.40
Cu	6.56	16.49	12.71
Cu	6.55	18.02	10.58
Cu	5.26	14.16	12.68
Cu	5.26	15.92	10.58
Cu	5.27	21.39	8.39
Cu	5.26	11.86	10.63
Cu	5.28	17.20	8.39
Cu	5.25	18.80	12.68
Cu	5.25	20.17	10.54
Cu	5.34	13.08	8.38
Cu	6.57	13.87	10.53
Cu	6.57	19.25	8.40
Cu	6.55	21.12	12.60
Cu	6.56	22.37	10.45
Cu	1.39	15.16	8.36
Cu	3.97	13.87	10.51
Cu	3.98	19.25	8.38
Cu	1.38	16.49	12.70
Cu	1.37	18.03	10.56
Cu	3.96	21.13	12.59
Cu	3.96	22.37	10.43
Cu	2.67	11.86	10.63
Cu	2.68	17.20	8.37
Cu	0.09	14.16	12.67
Cu	0.09	15.93	10.57
Cu	0.09	21.39	8.35
Cu	2.66	18.80	12.67
Cu	2.66	20.18	10.53
Cu	2.75	13.08	8.36
Cu	0.09	11.86	10.62
Cu	0.09	17.20	8.35
Cu	2.67	14.16	12.67
Cu	2.67	15.93	10.56
Cu	2.68	21.39	8.37
Cu	0.08	18.80	12.68

Cu	0.07	20.18	10.52
Cu	0.15	13.08	8.34
Cu	3.98	15.16	8.38
Cu	1.38	13.87	10.49
Cu	1.39	19.24	8.36
Cu	3.96	16.49	12.70
Cu	3.96	18.03	10.57
Cu	1.36	21.14	12.58
Cu	1.37	22.37	10.42
Cu	5.31	10.72	8.35
Cu	2.72	10.72	8.35
Cu	0.13	10.72	8.35
Cu	2.64	0.07	12.33
Cu	0.04	3.52	10.52
Cu	0.05	4.91	12.65
Cu	2.60	2.29	8.35
Cu	2.64	7.78	10.52
Cu	2.65	9.52	12.66
Cu	0.01	6.49	8.33
Cu	1.33	1.33	10.46
Cu	1.34	2.58	12.60
Cu	3.89	0.15	8.36
Cu	3.92	5.67	10.52
Cu	3.94	7.21	12.66
Cu	1.30	4.34	8.34
Cu	1.36	9.84	10.50
Cu	1.37	11.84	12.78
Cu	3.90	8.63	8.36
Cu	3.92	1.33	10.47
Cu	3.93	2.58	12.61
Cu	1.30	0.15	8.34
Cu	1.33	5.67	10.51
Cu	1.35	7.20	12.66
Cu	3.89	4.34	8.36
Cu	3.95	9.85	10.51
Cu	3.96	11.84	12.78
Cu	1.31	8.63	8.34
Cu	0.06	0.07	12.33
Cu	2.63	3.52	10.52
Cu	2.63	4.91	12.65
Cu	0.00	2.30	8.33
Cu	0.05	7.78	10.52
Cu	0.07	9.52	12.67
Cu	2.60	6.48	8.35
Cu	5.22	3.52	10.54

Cu	5.23	4.91	12.66
Cu	5.19	6.48	8.37
Cu	6.51	1.33	10.49
Cu	6.52	2.59	12.62
Cu	6.48	4.34	8.38
Cu	6.54	9.85	10.52
Cu	6.56	11.84	12.78
Cu	6.48	0.15	8.38
Cu	6.51	5.67	10.54
Cu	6.53	7.21	12.67
Cu	6.49	8.62	8.38
Cu	5.23	0.07	12.34
Cu	5.19	2.29	8.37
Cu	5.23	7.78	10.54
Cu	5.24	9.52	12.67

Table S5. Cartesian Coordination of the Cu∑5/(100) GB
Unit cell:

	x /Å	y /Å	z /Å
x	7.77	0.00	0.00
y	0.00	23.51	0.00
z	0.00	0.00	24.21

Atom	x /Å	y /Å	z /Å
Cu	13.48	10.37	5.15
Cu	15.59	11.68	5.34
Cu	13.49	10.41	9.00
Cu	15.54	0.04	8.79
Cu	17.84	1.18	5.29
Cu	17.83	1.18	8.86
Cu	14.36	2.28	5.35
Cu	13.41	4.57	5.25
Cu	12.23	1.00	9.07
Cu	14.30	2.26	8.66
Cu	13.45	4.56	8.95
Cu	16.65	3.50	5.30
Cu	16.63	3.47	8.85
Cu	20.11	2.40	5.28
Cu	22.36	3.64	5.24
Cu	20.10	2.40	8.87
Cu	22.36	3.66	8.92
Cu	25.02	4.79	5.59
Cu	18.95	4.70	5.29
Cu	21.20	5.92	5.28
Cu	18.95	4.69	8.86
Cu	21.21	5.92	8.86
Cu	23.45	7.12	5.21
Cu	2.17	3.66	5.24
Cu	2.17	3.67	8.92
Cu	4.43	2.44	5.29
Cu	4.43	2.43	8.87
Cu	3.33	5.95	5.29
Cu	3.31	5.95	8.86
Cu	5.58	4.74	5.31
Cu	5.58	4.73	8.87
Cu	6.71	1.25	5.31
Cu	8.95	0.06	5.35
Cu	6.71	1.24	8.87
Cu	9.00	0.08	8.84
Cu	11.11	10.53	5.27

Cu	11.12	10.47	8.86
Cu	7.87	3.59	5.30
Cu	10.11	2.35	5.33
Cu	11.04	4.61	5.17
Cu	7.90	3.55	8.87
Cu	10.18	2.34	8.75
Cu	11.06	4.61	8.98
Cu	12.21	1.16	4.63
Cu	15.53	5.84	5.35
Cu	15.57	5.83	8.85
Cu	17.80	7.02	5.29
Cu	17.83	7.02	8.88
Cu	12.27	6.81	5.13
Cu	14.35	8.09	5.44
Cu	12.28	6.80	9.11
Cu	14.35	8.09	8.79
Cu	16.61	9.31	5.33
Cu	16.62	9.34	8.87
Cu	1.03	7.12	8.89
Cu	20.09	8.24	5.29
Cu	22.36	9.50	5.24
Cu	20.10	8.23	8.87
Cu	22.35	9.48	8.92
Cu	25.00	10.67	5.60
Cu	18.92	10.52	5.31
Cu	21.19	0.07	5.29
Cu	18.94	10.52	8.86
Cu	21.19	0.07	8.87
Cu	23.46	1.24	5.25
Cu	1.08	7.12	5.21
Cu	2.20	9.51	5.22
Cu	2.13	9.51	8.90
Cu	4.44	8.27	5.30
Cu	4.41	8.28	8.88
Cu	3.35	0.10	5.29
Cu	3.33	0.09	8.86
Cu	5.62	10.57	5.33
Cu	5.60	10.57	8.87
Cu	6.71	7.08	5.32
Cu	9.00	5.96	5.36
Cu	6.69	7.07	8.89
Cu	8.98	5.92	8.85
Cu	7.92	9.36	5.34
Cu	7.92	9.38	8.88
Cu	10.25	8.17	8.72

Cu	23.49	7.09	8.89
Cu	25.03	4.83	8.54
Cu	10.26	8.20	5.52
Cu	1.06	1.26	5.25
Cu	25.04	10.64	8.55
Cu	23.46	1.25	8.92
Cu	1.07	1.26	8.92
Cu	13.56	0.43	7.08
Cu	16.13	1.73	7.07
Cu	18.40	2.94	7.07
Cu	12.21	3.05	7.13
Cu	14.96	4.03	7.08
Cu	13.69	6.33	7.12
Cu	17.26	5.24	7.08
Cu	20.68	4.16	7.07
Cu	22.95	5.39	7.06
Cu	19.51	6.46	7.07
Cu	21.76	7.67	7.07
Cu	1.58	5.40	7.06
Cu	3.85	4.18	7.09
Cu	2.76	7.69	7.07
Cu	5.00	6.50	7.10
Cu	6.14	2.99	7.09
Cu	8.41	1.79	7.08
Cu	10.82	0.61	7.05
Cu	7.28	5.31	7.10
Cu	9.58	4.10	7.08
Cu	10.87	6.33	7.11
Cu	16.12	7.53	7.11
Cu	18.37	8.76	7.09
Cu	12.33	8.78	7.09
Cu	14.93	9.83	7.11
Cu	17.25	11.07	7.10
Cu	0.67	8.94	7.03
Cu	20.67	9.99	7.08
Cu	22.94	11.22	7.09
Cu	19.52	0.61	7.08
Cu	21.77	1.82	7.08
Cu	1.59	11.24	7.06
Cu	3.86	10.02	7.09
Cu	2.77	1.84	7.08
Cu	5.03	0.65	7.09
Cu	6.14	8.82	7.11
Cu	8.42	7.62	7.12
Cu	7.30	11.14	7.11

Cu	9.57	9.96	7.10
Cu	23.85	8.93	7.08
Cu	25.02	6.79	7.03
Cu	25.02	0.95	7.09
Cu	23.85	3.09	7.09
Cu	0.68	3.09	7.08

References

[1] C. Dong, J. Fu, H. Liu, T. Ling, J. Yang, S. Z. Qiao, X.-W. Du, *J. Mater. Chem. A* **2017**, *5*, 7184-7190.

[2] X. Feng, K. Jiang, S. Fan, M. W. Kanan, *J. Am. Chem. Soc.* **2015**, *137*, 4606-4609.

[3] G. Kresse, & Furthmüller, *J. Phys. Rev. B* **1996**, *54*, 11169–11186.

[4] J. Wellendorff, K. T. Lundgaard, A. Møgelhøj, V. Petzold, D. D. Landis, J. K. Nørskov, T. Bligaard, K. W. Jacobsen, *Phy. Rev.B* **2012**, *85*, 235149.

[5] G. Kresse, D. Joubert, *Phy.l Rev. B* **1999**, *59*, 1758-1775.

[6] J. K. Norskov, J. Rossmeisl, A. Logadottir, L. Lindqvist, J. R. Kitchin, T. Bligaard, H. Jonsson, *J. Phys. Chem. B* **2004**, *108*, 17886-17892.

[7] S. Hanselman, M. T. M. Koper, F. Calle-Vallejo, *ACS Energy Lett.* **2018**, *3*, 1062-1067.

[8] X. Liu, J. Xiao, H. Peng, X. Hong, K. Chan, J. K. Norskov, *Nat .Commun.* **2017**, *8*, 15438.

[9] R. Nelson, C. Ertural, J. George, V. L. Deringer, G. Hautier, R. Dronskowski, *J. Comput. Chem.* **2020**, *41*, 1931-1940.
